# Strain-Level Discrimination of Shiga Toxin-Producing *Escherichia coli* in Spinach Using Metagenomic Sequencing

**DOI:** 10.1371/journal.pone.0167870

**Published:** 2016-12-08

**Authors:** Susan R. Leonard, Mark K. Mammel, David W. Lacher, Christopher A. Elkins

**Affiliations:** Division of Molecular Biology, Center for Food Safety and Applied Nutrition, U.S. Food and Drug Administration, Laurel, Maryland, United States of America; Cornell University, UNITED STATES

## Abstract

Consumption of fresh bagged spinach contaminated with Shiga toxin-producing *Escherichia coli* (STEC) has led to severe illness and death; however current culture-based methods to detect foodborne STEC are time consuming. Since not all STEC strains are considered pathogenic to humans, it is crucial to incorporate virulence characterization of STEC in the detection method. In this study, we assess the comprehensiveness of utilizing a shotgun metagenomics approach for detection and strain-level identification by spiking spinach with a variety of genomically disparate STEC strains at a low contamination level of 0.1 CFU/g. Molecular serotyping, virulence gene characterization, microbial community analysis, and *E*. *coli* core gene single nucleotide polymorphism (SNP) analysis were performed on metagenomic sequence data from enriched samples. It was determined from bacterial community analysis that *E*. *coli*, which was classified at the phylogroup level, was a major component of the population in most samples. However, in over half the samples, molecular serotyping revealed the presence of indigenous *E*. *coli* which also contributed to the percent abundance of *E*. *coli*. Despite the presence of additional *E*. *coli* strains, the serotype and virulence genes of the spiked STEC, including correct Shiga toxin subtype, were detected in 94% of the samples with a total number of reads per sample averaging 2.4 million. Variation in STEC abundance and/or detection was observed in replicate spiked samples, indicating an effect from the indigenous microbiota during enrichment. SNP analysis of the metagenomic data correctly placed the spiked STEC in a phylogeny of related strains in cases where the indigenous *E*. *coli* did not predominate in the enriched sample. Also, for these samples, our analysis demonstrates that strain-level phylogenetic resolution is possible using shotgun metagenomic data for determining the genomic relatedness of a contaminating STEC strain to other closely related *E*. *coli*.

## Introduction

Many foodborne outbreaks have been attributed to contaminated fresh leafy vegetables [1–4]. Of importance, 46% of the total hospitalizations due to leafy vegetable-associated outbreaks between 1973 and 2012 were caused by Shiga toxin-producing *Escherichia coli* (STEC) [3]. During this time period, consumption of fresh bagged spinach contaminated with STEC led to six deaths and over 100 hospitalizations. This high rate of hospitalization is the result of severe complications such as hemorrhagic colitis and hemolytic-uremic syndrome that STEC infection causes in some individuals [5]. Data taken from microbial surveys of fresh produce samples over a ten year period identified spinach as the produce item most often contaminated with STEC, with an estimated prevalence rate of 0.5 to 0.6% [4]. The infectious dose for STEC has been estimated to be 10 to 100 CFU [6–8], thus methods for detecting STEC in foods require a high degree of sensitivity.

Partial characterization of an STEC strain associated with fresh produce is an important part of the detection procedure since not all STEC strains are considered to be pathogenic to humans and, hence, pose a regulatory food safety concern. STEC genomes possess at least one allele variant of either the *stx1* or *stx2* genes encoding Shiga toxin [5] with particular variants linked to serious human illness, namely, *stx1a*, *stx2a*, *stx2c*, and *stx2d* [9]. In addition to the Shiga toxin alleles, the presence of the genes encoding intimin, *eae*, and enterohemolysin, *ehxA*, is considered an indicator of pathogenicity [2, 4, 10]. Furthermore, a detection method that incorporates the ability to simultaneously determine the presence/absence of multiple *E*. *coli* virulence genes would be ideal since hybrid pathotype strains involving STEC have been reported to cause severe disease [11–14]. Along with specific virulence genes, the serotype of a contaminating STEC strain is considered when evaluating pathogenicity as some are associated with a history of disease. For example, STEC O157:H7 has been recognized and designated as a human pathogen for over 30 years but illness due to non-O157 STEC is becoming increasingly common, and non-O157 STEC has been isolated from many food commodities including produce [2, 15]. The serogroups other than O157 reportedly associated with human illness most often are O26, O45, O103, O111, O121, and O145 [15], however less common serogroups such as O91, O104, and O113 have also been known to cause severe illness [4, 10, 11].

We have demonstrated that shotgun metagenomics can be utilized to detect the presence of *E*. *coli* contamination in fresh leafy produce within a complex microbial background while simultaneously detecting multiple virulence genes as well as the molecular somatic O and flagellar H types [16]. Ideally, shotgun metagenomic sequencing data would also be used to determine the genomic relatedness of a contaminating STEC strain to STEC isolated from other samples for epidemiological/clinical relevance in track-and-trace pursuits. In our previous work, a discriminative *k*-mer signature bioinformatic scheme was developed for microbial community analysis that includes classification of *E*. *coli* at the phylogroup level [16]. However, significantly increased phylogenetic resolution would be attained utilizing *E*. *coli* core gene single nucleotide polymorphism (SNP) analysis of metagenomic data. Our earlier experiments revealed that a sample enrichment step is necessary to detect low STEC contamination levels in spinach (0.1 CFU/g) by metagenomic profiling due to the high level of indigenous microbial load[16]. That work was limited to STEC O157:H7 strain Sakai for spiking, however, it is expected that different STEC strains will grow at different rates and to different population abundances during the enrichment process [17].

As an extension to previous work, determining the detection sensitivity using a variety of STEC strains is crucial to evaluating the robustness of this approach. In the present work, detection of STEC in spinach was assessed using STEC strains for spiking that are relevant to human illness and vary in *E*. *coli* phylogroup, serotype, and Shiga toxin allele subtype. Sample reproducibility and the impact of the indigenous microbiota were examined. Our results demonstrate that, provided indigenous *E*. *coli* is not present in the spinach, it is possible to achieve strain-level phylogenetic resolution using shotgun metagenomic data to discriminate contaminating STEC strains from other *E*. *coli* strains.

## Materials and Methods

### STEC strains

The STEC strains used for spiking experiments in this study are listed in Table 1. The STEC strains EC1660, EC1705, EC1917, and EC2002 were sequenced as part of this work. The genomes of the remaining eight STEC strains were obtained from GenBank (Table 1). All of the strains are clinical isolates except EC1738, which is a food isolate. Two hybrid strains were included in the study: EC1894 is a hybrid STEC/enteroaggregative pathotype strain and EC2634 is a hybrid STEC/enterotoxigenic pathotype strain. As such, EC1894 and EC2634 possess the virulence genes *aggR* and *stb*, respectively.

**Table 1:** Characteristics of STEC strains included in this study.

**Strain**	**Pseudonyms**	**Serotype**	**Phylogroup**	**Shiga toxin(s)**	**Source**	**Accession no.**
EC1276	Sakai	O157:H7	E	Stx1a, Stx2a	Japan, sprout outbreak, 1996	BA000007
EC1611	MI06-63, TW14359	O157:H7	E	Stx2a, Stx2c	USA (MI), spinach outbreak, 2006	CP001368
EC1738	550659	O157:H7	E	Stx2c	USA, cookie dough outbreak, 2009	AKMN01
EC1971	MI10-01, TW16058	O145:H28	E	Stx2a	USA (MI), romaine lettuce outbreak, 2010	JHEZ01
EC2002	0202 2501, TW14998	O145:H28	E	Stx1a, Stx2a	USA (CT), clinical isolate, 2004	SRS1263853
EC1660	MT#2, TW08023	O121:H19	B1	Stx2a	USA (MT), clinical isolate, 1998	SRS1263852
EC1705	EC-6804, TW08980	O121:H19	B1	Stx2a	Japan, well water (clinical isolate), 2002	SRS1263851
EC1917	2012000615	O26:H11	B1	Stx1a	USA (OH), clover sprout outbreak, 2012	SRS1263855
EC1894	2011C-3493	O104:H4	B1	Stx2a	Germany, sprout outbreak, 2011	CP003289
EC2634	B2F1	O91:H21	B1	Stx2d1, Stx2d2	Canada, patient with HUS, 1985	AFDQ01
EC1623	CL-3, TW01391	O113:H21	B1	Stx2a, Stx2d	Canada, patient with HUS, 1980	AGTH01
EC2635	C165-02	O73:H18	D	Stx2d	Patient with bloody diarrhea	AFDR01

### DNA isolation and whole genome sequencing of STEC strains

Genomic DNA was extracted from overnight cultures grown in Luria broth at 37°C using the DNeasy Blood and Tissue Kit (Qiagen, Germantown, MD, USA). Sequencing libraries were prepared from genomic DNA with the Nextera DNA Sample Preparation Kit (Illumina, San Diego, CA, USA) and sequenced on the Illumina MiSeq Platform, generating paired-end 250 bp reads in sufficient quantity to provide between 104X and 137X coverage for each genome. Raw reads were trimmed and draft genome sequences were assembled *de novo* with CLC Genomics Workbench v8.5.1 (CLC bio, Boston, MA, USA).

### Metagenomic sample processing and sequencing

Packages of ready-to-eat spinach (227 g) were purchased from a grocery store and stored at 4°C until use. Triplicate biological spiked spinach samples were prepared for each of the 12 STEC strains. The spinach was purchased over a period of 3 months so that the spinach used for any particular STEC strain was purchased at different times. Each bag of spinach was divided and used for two spike experiments using different STEC strains. STEC strains were grown overnight in modified Buffered Peptone Water with pyruvate (mBPWp) media. The overnight culture was diluted appropriately to add, depending on the strain, between 270 and 660 μL containing the target number of spiked cells for a spike level of 0.1 CFU/g spinach. Dilutions of the overnight culture were spread on Luria Bertani plates that were incubated overnight at 37°C to verify the level of artificial contamination of the spinach. Experiments were carried out using a slightly modified version of the BAM protocol [18]. Briefly, 1627 mL filter whirlpak bags containing 100 g spinach were spiked with the appropriate STEC strain and 225 mL mBPWp was added. Bags were manually massaged and then shaken at 37°C and 185 rpm for 30 min, followed by a static incubation at 37°C for 4.5 hrs. Three antimicrobials: acriflavin, cefsulodin, and vancomycin, were added to the samples to final concentrations of 10 mg/L, 10 mg/L, and 8 mg/L, respectively, and the bags were manually massaged again to distribute the supplements. This was followed by a static incubation at 42°C for 18 h, after which the bags were manually massaged and 1 or 2 mL aliquots were removed and pelleted. All pellets were stored at -20°C prior to gDNA extraction. Genomic DNA was extracted from the metagenomic samples using the DNeasy Blood and Tissue Kit (Qiagen) and sequencing libraries were prepared with the Nextera DNA Sample Preparation Kit (Illumina). Twelve samples were multiplexed and sequenced on the Illumina MiSeq Platform, generating paired-end 250 bp reads. The number of reads per sample averaged 2,393,792 and ranged from 1,248,638 to 5,189,667.

### Microbial community analysis

Metagenomic analysis for microbial composition was conducted using custom C++ programs developed to compile a *k*-mer signature database containing multiple unique 25 bp sequences per target entry (bacterial species, phylogroup, or serovar) and then identify bacterial species in a sample by the unique 25-mers using FASTQ files as input. Determination of database entries was based on previous work [16], BLAST results using some sequence reads generated in this work as queries, and results from several of the samples utilizing MetaPhlAn version 2.0.0 [19]. For each species of interest, each non-duplicated 25-mer from a reference whole genome sequence was placed into a database. The *k*-mers not found in at least 2/3 of a set of additional genome sequences of the same species as well as *k*-mers found in genomes of other species were removed. The resulting *k*-mer database used in this work contains 352 target entries, each consisting of approximately 40,000 (range 255 to 80,000) unique *k*-mers. The coverage of each species’ 25-mer set was determined by testing each possible 100-bp read in a reference genome against the 25-mer set and tallying the number of reads in the genome that are matched by a 25-mer. The database includes 84 different bacterial genera and can differentiate between *E*. *coli* phylogroups A, B1, B2, D, and E. An attempt to identify each read from the sequencing run was made by matches to the 25-mer database. Normalization was performed to correct for bias due to differing number of *k*-mers used per database entry and genome size, and the results are tabulated as percent contribution to the microbial population of identified species for each database entry. Using this method, unspiked spinach samples enriched 23 hours containing no indigenous *E*. *coli* as inferred by molecular serotyping were determined to have an average *E*. *coli* background relative abundance of 0.01%.

### Read mapping, molecular serotyping, and assembly of metagenomic sequence data

Raw shotgun metagenomic sequence reads were trimmed and mapped to the respective STEC genome using CLC Genomics Workbench v8.5.1 (CLC bio). Mapping parameters used were 98% identity for 100% of the trimmed read length. Using unspiked spinach samples containing no indigenous *E*. *coli* to obtain a background value, the percent reads mapping to the STEC Sakai genome averaged 0.23%. The number of trimmed reads mapping to several genes involved in adhesion (*afaD*, *aggR*, *bfpA*, *eae*, *papDJK*, *perA*, *saa*), invasion (*icsP*, *mkaD*, *ospB*), and toxins (*cdtB*, *cnf*, *ehxA*, *hlyA*, LT, ST, *stx1*, *stx2*, *subAB*), as well as the *wzm*, *wzt*, *wzx*, *wzy*, *fliC*, *flkA*, *fllA*, *flmA*, and *flnA* molecular serotyping loci [20–25] was determined using SeqMan NGen v12.2.0 with the default reference-based assembly parameters for Illumina reads (DNASTAR Inc, Madison, WI, USA). The trimmed shotgun metagenomic sequence data for each sample was assembled *de novo* using CLC Genomics Workbench v8.5.1 (CLC bio) and filtered to retain contigs >500 bp and with >5x coverage. For five of the samples, the shotgun metagenomic sequence reads were mapped using Bowtie 2 [26] to the genomes of 329 diverse *E*. *coli* defining major genotypes of the entire *E*. *coli* chromosomal landscape. Reads that were paired matches to at least one genome (referred to as *E*. *coli* specific reads) were used in a *de novo* assembly using CLC Genomics Workbench v9.0.1 (CLC bio) and filtered to retain contigs >200 bp and with >5x coverage. The trimmed reads for these five samples were also mapped to the *E*. *coli* K-12 MG1655 genome (GenBank accession number U00096.3) using CLC Genomics Workbench v9.0.1 with mapping parameters of 95% identity for 95% of the trimmed read length. The consensus sequence was extracted and the *E*. *coli* K-12 MG1655 reference-based assemblies were used for SNP analysis.

### Whole genome phylogeny and SNP analysis

Using the *E*. *coli* K-12 MG1655 genome as a reference, BLASTn analysis of 42 representative closed *E*. *coli* / *Shigella* genomes identified 2542 conserved chromosomal genes present in at least 41 of the genomes with at least 95% sequence similarity to K-12 MG1655. Strain relatedness was determined through phylogenetic analysis of sequence variation of these 2542 conserved chromosomal genes. Sequences for these genes were extracted from the genomes of the 12 STEC strains, 36 metagenomic assemblies, 5 *E*. *coli* specific reads assemblies, and 5 *E*. *coli* K-12 MG1655 reference-based assemblies via BLASTn searches using the sequences from K-12 MG1655 as the queries, and for each genome or assembly, the matching bases were aligned in one file for each core gene. Each core gene alignment was then scanned for nucleotide variation at each position. A total of 876,716 polymorphic sites were identified in 4822 available genomic sequences (data not shown) and the number of sites missing from each genome, metagenomic assembly, *E*. *coli* specific reads assembly, or reference-based assembly was tabulated. For the remaining SNP sites that the genome and metagenomic assembly for the spiked STEC strain had in common, the number of nucleotide mismatches was counted. Similarly, the number of mismatches at SNP positions between the spiked STEC genome and the *E*. *coli* specific assembly or K-12 MG1655 reference-based assembly resulting from the spiked sample was determined. For the phylogeny, closed or draft genomes of reference STEC and *E*. *coli* prototype strains from other *E*. *coli* pathotypes were included in the phylogenetic analysis for comparative purposes. A total of 206,312 polymorphic sites were identified among the genomes which were then concatenated and imported into MEGA 3.1 [27] for neighbor-joining analysis using a *p* distance matrix and 500 bootstrap replications with gaps in the data handled by the pairwise deletion option.

### Nucleotide sequence accession numbers

The sequence reads for the draft genomes of the STEC strains EC1660, EC1705, EC1917, and EC2002 were deposited in the NCBI Sequence Read Archive (SRA) database under accession nos. SRS1263852, SRS1263851, SRS1263855, and SRS1263853, respectively (Table 1). Metagenomic sequence data from this study was deposited in the SRA under the study accession no. SRP083779.

## Results

### Bacterial community analysis

**Fig 1:**
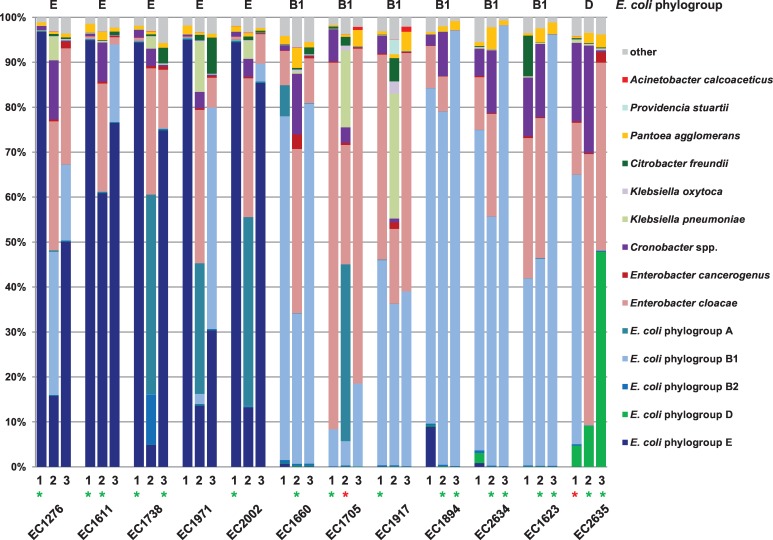
Microbial community associated with enriched STEC-spiked spinach. Spinach samples were spiked with STEC at a level of 0.1 CFU/g and enriched. Triplicate experiments for each strain were performed using bagged spinach purchased at different times and designated m1, m2, and m3. The *E*. *coli* phylogroup of the spiked STEC strain is indicated at the top of the figure. Bacterial genera or species contributing ≥1% to the total identified population in at least one sample are shown and the sum of all other species identified in lower abundances is included in “other”. Samples in which the serogroup of the spiked STEC was the only serogroup detected (green asterisk) and samples for which the serotype or virulence genes were not detected (red asterisk) are indicated.

Shotgun metagenomic sequencing was performed on samples of spiked fresh ready-to-eat bagged spinach. The spinach was spiked at a level of 0.1 CFU/g with one of 12 different STEC strains representing eight serotypes that had been either associated with an outbreak or caused severe illness in humans (Table 1). The spiked samples were enriched for 23 hrs. Three independent experiments (designated m1, m2, and m3) were performed for each of the 12 STEC strains using spinach of the same brand but purchased at different times. The relative abundances of bacterial species present in the enriched spiked spinach samples were determined (Fig 1). *E*. *coli* was classified at a subspecies level, namely with abundances of the *E*. *coli* phylogroups A, B1, B2, D, and E. This was particularly valuable since indigenous *E*. *coli* was found in many of the spinach samples (see Molecular serotyping section below). The *E*. *coli* phylogroup of the spiked STEC strain was observed in all 36 samples and could be unambiguously determined for samples in which the spiked STEC strain was the only *E*. *coli* strain present. The community analysis results reveal the predominant phylogroups of the indigenous *E*. *coli* strains to be A and B1; however B2, D, and E were also observed in at least one sample each (Fig 1). Whether the spiked STEC strain or a combination of the spiked strain and indigenous *E*. *coli* strain(s) were present in the samples, *E*. *coli* was the predominant species present after enrichment. The second most abundant species in 75% of the samples was *Enterobacter cloacae*. Combinations of other Enterobacteriaceae including *Cronobacter*, *Citrobacter*, and *Klebsiella* species as well as *Pantoea agglomerans* comprised the majority of the remainder of the bacterial communities. Enrichment complexities were fairly similar, with a range of 0 to 4 genera (average 2) considering bacterial genera other than *Escherichia* contributing ≥1% relative abundance to the total identified population. Below this level (≥0.1% relative abundance), the complexity was still low with an average of 4 genera (range 1 to 6).

Variation in the percent abundance of the spiked STEC strain was observed among the three biological replicates for a given strain and even between those replicate samples in which no indigenous *E*. *coli* was detected (Fig 1). The STEC strains in samples EC1705m1 and EC2635m2 have the lowest percent abundance for samples in which the spiked strain was the only *E*. *coli* detected. The percent relative abundance of the spiked STEC and indigenous *E*. *coli* strain(s) that belong to different phylogroups can be observed in the community analysis results (Fig 1). However, when both the spiked STEC and indigenous *E*. *coli* strain(s) belong to the same phylogroup (B1 in four samples, namely, EC1623m1, EC1705m3, EC1917m2, and EC1917m3), the percent abundance of the spiked STEC cannot be accurately determined from the community analysis alone.

### Molecular serotyping of *E*. *coli* in metagenomic samples

Both the *E*. *coli* O and H types present in the enriched spiked STEC samples were determined from the shotgun metagenomic data. For most samples, there were a sufficient number of reads mapping to the respective serotyping loci to confidently determine the presence of the spiked strain (Table 2). However, the molecular serotype of the spiked STEC strain could not be detected with certainty in 2 samples, namely EC1705m2 and EC2635m1. Sample EC1705m2 had only three reads mapping to the O121 *wzx* allele, and no reads mapping to the O121 *wzy* allele. Furthermore, the H19 *fliC* allele was not detected in the sequence data (Table 2). However, *E*. *coli* serogroups O25 and O18, along with H types H34 and H7, were identified in sample EC1705m2. These *E*. *coli* O25:H34 and O18:H7 strains (O and H type combinations determined by a comparison of read counts mapping to the respective serotyping loci) undoubtedly represent indigenous *E*. *coli* in the bagged spinach that grew to a greater abundance than the spiked strain during enrichment. Similarly, there were insufficient reads mapping to the appropriate O and H loci (O73:H18) to confidently determine their presence for sample EC2635m1 (Table 2). In this case, *E*. *coli* serogroups O26 and O166 were identified along with H types H16, H15, and H11. In fact, the serogroup of the spiked STEC strain was the single *E*. *coli* serogroup detected in only 17/36 (47%) of the samples.

**Table 2:** Read counts mapping to virulence genes and serotyping loci.

**Sample**^a^	***stx1a***	***stx2a***	***stx2c***	***stx2d***	***eae***	***ehxA***	***wzx***^b^	***wzy***^b^	***fliC***^b^
EC1276m1*	807	1026	0	0	2153	1189	728	532	674
EC1276m2	29	31	0	0	82	36	39	30	71
EC1276m3	285	297	0	0	852	448	334	220	314
EC1611m1*	*49*	558	537	0	1461	685	470	382	409
EC1611m2*	0	307	288	0	912	322	282	223	273
EC1611m3	0	405	404	0	1074	439	276	230	386
EC1738m1*	0	0	510	0	1318	484	376	326	386
EC1738m2	0	0	27	0	76	38	27	26	29
EC1738m3*	0	0	313	0	1104	420	189	185	271
EC1971m1	0	537	0	0	1371	490	280	219	231
EC1971m2	0	69	0	0	199	51	14	18	24
EC1971m3	0	182	0	0	516	198	77	74	78
EC2002m1*	474	374	0	0	1340	593	251	186	194
EC2002m2	119	119	0	0	429	150	72	69	61
EC2002m3	441	294	0	0	1185	682	154	143	197
EC1660m1	0	444	0	0	902	104	400	284	229
EC1660m2*	0	236	0	0	512	172	186	106	131
EC1660m3	0	199	0	0	414	254	223	143	111
EC1705m1*	0	112	0	0	233	118	103	85	59
EC1705m2	0	2	0	0	3	0	3	0	0
EC1705m3	0	35	0	0	70	51	23	19	13
EC1917m1*	190	0	0	0	428	78	73	74	90
EC1917m2	24	0	0	0	77	18	46	31	22
EC1917m3	79	0	0	0	246	65	33	53	19
EC1894m1	*4*	139	0	0	*10*	0	149	79	24
EC1894m2*	0	213	0	0	0	0	280	221	50
EC1894m3*	0	999	0	0	0	0	979	669	195
EC2634m1	*72*	0	0	373	*315*	616	112	126	84
EC2634m2*	0	0	0	354	0	377	190	179	50
EC2634m3*	0	0	0	1991	0	2157	579	593	304
EC1623m1	0	86	0	77	0	221	62	54	21
EC1623m2*	0	151	0	148	0	282	116	102	59
EC1623m3*	0	507	0	510	0	875	395	247	155
EC2635m1	*16*	*1*^c^	*1*^c^	1^c^	*57*	*28*	3	2	4
EC2635m2*	0	0	0	24	0	0	12	32	17
EC2635m3*	0	0	0	270	0	0	140	140	146

Values shown in grey italic font are not expected for the spiked STEC strain. Locus query lengths are: *stx1* (1227 bp), *stx2* (1182 bp), *eae* (2805–2847 bp), *ehxA* (2997 bp), *wzx* (1236–1395 bp), *wzy* (1023–1332 bp), *fliC* (204–678 bp)

^a^ Samples for which the spiked strain serogroup was the only serogroup detected are marked *.

^b^ Allele specific for the serotype of the spiked STEC strain.

^c^ The read mapped to a location that is identical among the *stx2a*, *stx2c*, and *stx2d* subtypes, so the actual subtype present in this sample could not be determined, but was expected to be *stx2d* since that is the allele strain EC2635 possesses.

The molecular serotyping results determined an indigenous *E*. *coli* prevalence of 61% (11/18 bags), with 8 bags containing more than one indigenous *E*. *coli* serogroup. Regarding serogroup diversity identified in the metagenomic samples, O types (other than that of the spiked strain) included O9, O18, O25, O26 (samples EC2634m1 and EC2635m1), O29, O37, O45, O101, O112, O120, O166, O171, and O187. As an example, sample EC1276m3 contained reads matching the O45 *wzx* and *wzy* alleles as well as the O101 *wzm* and *wzt* alleles in addition to the expected O157 *wzx* and *wzy* loci. Indigenous *E*. *coli* H types identified (other than those associated with any of the spiked strains) included H12, H14, H15, H16, H34, H35, and H40. The relative abundance of the spiked STEC compared to the indigenous *E*. *coli* strain(s) could be estimated by the number of reads mapping to the respective O and H loci. Analysis of the mapping results revealed that the spiked strain grew to a greater abundance than the indigenous strain(s) in 9 samples (EC1276m3, EC1611m3, EC1623m1, EC1660m1, EC1660m3, EC1894m1, EC1971m1, EC2002m3, and EC2634m1), while the indigenous strain(s) outgrew the spiked strain in the remaining 10 samples.

Read counts mapping to the serotyping loci of the spiked STEC reported in Table 2 contain both the forward and reverse reads from the paired-end data. However, sequencing only one 250 bp read would reduce the sequencing time. The molecular serotype of the three samples with the fewest number of reads mapping to the correct serotyping loci, EC1705m3, EC1971m2, and EC2635m2 (Table 2), could still be detected using only the forward reads.

### Virulence gene identification

Shiga toxin allele subtype and presence/absence of *eae* and *ehxA* are used along with molecular serotype to assess pathogenicity potential for humans, and thus are of regulatory concern. In all samples except EC1705m2 and EC2635m1, there were sufficient reads mapping to virulence gene loci to identify *eae* and *ehxA* in the respective spiked STEC strains (Table 2). Furthermore, the correct Shiga toxin gene allele subtype(s) were determined even for those samples spiked with a strain possessing two *stx* subtypes. Although not every read could be unambiguously assigned to a particular *stx2* allele subtype, reads covering the regions with discriminatory SNPs were present, thus the correct subtype could be identified. As with molecular serotyping, virulence gene identification was also possible using only the forward 250 bp sequence read. The genomes of STEC strains EC1623, EC1894, EC2634, and EC2635 do not possess *eae*, and the genomes of STEC strains EC1894 and EC2635 do not possess *ehxA*. The virulence gene profiles are consistent with this fact and contain no false positives for samples in which the spiked STEC strain was the only serogroup detected (Table 2).

In some samples where indigenous *E*. *coli* strains were detected by molecular serotyping, virulence genes were observed that are not contained in the genome of the spiked strain. In particular, *stx1a* and *eae* were detected in samples EC1894m1, EC2634m1, and EC2635m1. It is noteworthy that the spinach for samples EC1894m1 and EC2634m1 was taken from the same bag, while a different bag of spinach was used for sample EC2635m1. Along with the virulence genes included in Table 2, genes defining other *E*. *coli* pathotypes were queried for in the analysis. The *aggR* gene was detected in all three samples spiked with STEC EC1894 as expected (48, 124, and 560 reads), and the heat-stable enterotoxin *stb* gene was detected in samples EC2635m2 and EC2635m3 as expected (15 and 84 reads).

### Genome coverage

In addition to characterization using molecular serotyping and detection of virulence genes, we sought to determine whether strain level identification could be performed using our metagenomic datasets. The microbial community analysis provides an estimate of the relative abundance of the spiked STEC strain, but not the genome coverage. To corroborate the percent abundance results and to assess average genome coverage, the trimmed shotgun metagenomic sequence reads were mapped to the respective spiked STEC genome for the 17 samples in which the spiked STEC was the only *E*. *coli* present in the enriched sample. The percent total reads mapping to the STEC genome was found to vary among samples and even between samples using the same spiked strain (Table 3). The average genome coverage ranges from 5x to 145x, with four samples having coverages less than 30x, of which only two, EC1705m1 and EC2635m2, have a coverage below 20x (Table 3). Therefore, we reasoned that there is sufficient genome coverage to perform *E*. *coli* core gene SNP analysis on most samples.

**Table 3:** Percent reads mapping to STEC genome and genome coverage.

Sample^a^	Mapped reads (%)	Average genome coverage (x)
EC1276m1	81.2	100
EC1611m1	77.1	67
EC1611m2	42.6	50
EC1738m1	72.1	55
EC1738m3	48.3	48
EC2002m1	81.0	59
EC1660m2	39.7	32
EC1705m1	15.4	16
EC1917m1	35.7	24
EC1894m2	54.7	38
EC1894m3	87.5	145
EC2634m2	39.2	35
EC2634m3	89.6	137
EC1623m2	32.2	28
EC1623m3	83.9	74
EC2635m2	5.56	5
EC2635m3	31.5	35

^a^ Only samples in which the spiked STEC strain was the only *E*. *coli* detected are included.

### SNP analysis and strain-level phylogeny

**Fig 2:**
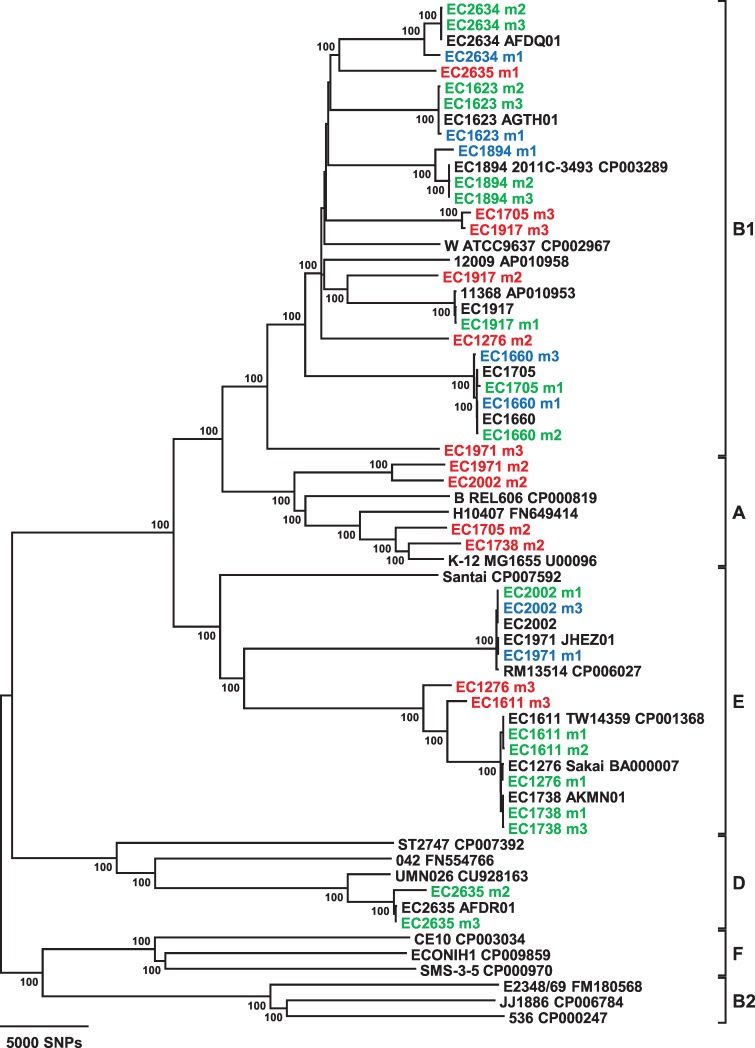
Phylogenetic relationships resulting from *E*. *coli* core gene SNP analysis of spiked STEC genomes, metagenomic samples, and other *E*. *coli* strains. This neighbor-joining tree was constructed using a *p* distance matrix and 500 bootstrap replications. Metagenomic samples are color-coded as follows: green, spiked STEC only *E*. *coli* detected in sample; blue, indigenous *E*. *coli* present but clusters with spiked strain; and red, indigenous *E*. *coli* present and does not cluster with spiked strain. Closed genome strains are labeled with their strain name and GenBank accession number. Phylogroups are indicated by the square brackets. Bootstrap values >80% are given at the internal nodes.

When testing spinach samples for STEC contamination, no *a priori* knowledge of the possible contaminating strain is available. Thus, rather than use the reference based assemblies generated from mapping reads to the spiked strain genome, sequence data from the samples was assembled *de novo* and *E*. *coli* core gene SNP analysis was performed on the resulting metagenomic contigs. Of the 876,716 SNP sites identified for use in the *E*. *coli* SNP analysis scheme (see Materials and Methods), those covered in common between the spiked STEC genome and the assembled metagenome for each sample were determined (Table 4). Next, the nucleotide mismatches at the shared SNP sites was tabulated (Table 4). While the number of mismatches totaled over 2000 for 12 samples, the number of mismatches was less than 20 for 14 of the samples. As expected, the number of mismatches was lowest for those samples in which the spiked STEC was the only *E*. *coli* detected or the abundance of the indigenous *E*. *coli* was much lower than that of the spiked strain (Fig 1, Table 4). A phylogenetic tree based on *E*. *coli* core gene SNP analysis was constructed that includes the spiked STEC genomes, the 36 metagenomic sample assemblies, and representative genomes from the different *E*. *coli* phylogroups (Fig 2). The results reveal that on this coarse scale, the metagenomic sample and genome of the spiked STEC strain cluster together when the spiked strain is the only *E*. *coli* present (17 samples) and even in some cases where there was an indigenous *E*. *coli* strain in the spinach sample (7 samples). For the remaining 12 samples, the abundance of the indigenous *E*. *coli* compared to the spiked strain caused the metagenomic sample to be placed outside the cluster containing the spiked STEC strain genome and even in a different phylogroup in some cases.

**Table 4:** Number of contigs, single nucleotide polymorphic (SNP) sites and nucleotide mismatches at SNP sites.

**Strain**	**contigs**^a^	**Covered SNPs m1**^b^	**Mismatches m1**^c^	**contigs**^a^	**Covered SNPs m2**^b^	**Mismatches m2**^**c**^	**contigs**^a^	**Covered SNPs m3**^b^	**Mismatches m3**^c^
EC1276	327	873248	15	7583	844403	32842	1801	818245	4710
EC1611	948	873445	0	4908	872810	43	1314	851317	4077
EC1738	1230	871720	0	8212	840233	33899	3519	871667	10
EC1971	228	874165	0	7091	836773	26915	5973	803805	22507
EC2002	193	873248	11	10861	806822	29025	1475	873252	11
EC1660	312	875599	0	4805	875394	21	881	868009	217
EC1705	4756	868669	142	6324	872445	25311	7590	861015	19182
EC1917	2018	873292	47	4947	847663	11072	4784	856966	15496
EC1894	2902	853263	1743	2480	876238	14	469	876620	1
EC2634	7299	825772	1713	4703	875152	15	126	875165	2
EC1623	3964	867138	118	4625	872914	64	158	873595	1
EC2635	4189	851205	45934	7509	511437	1134	278	871661	3

^a^ Number of contigs in the metagenome (filtered to retain contigs >500 bp and >5x coverage)

^b^ Number of SNP locations covered in common between the spiked STEC genome and the assembled metagenome out of 876,716 included in the core gene analysis scheme.

^c^ Values for samples in which the spiked STEC was the only *E*. *coli* detected are underlined.

**Fig 3:**
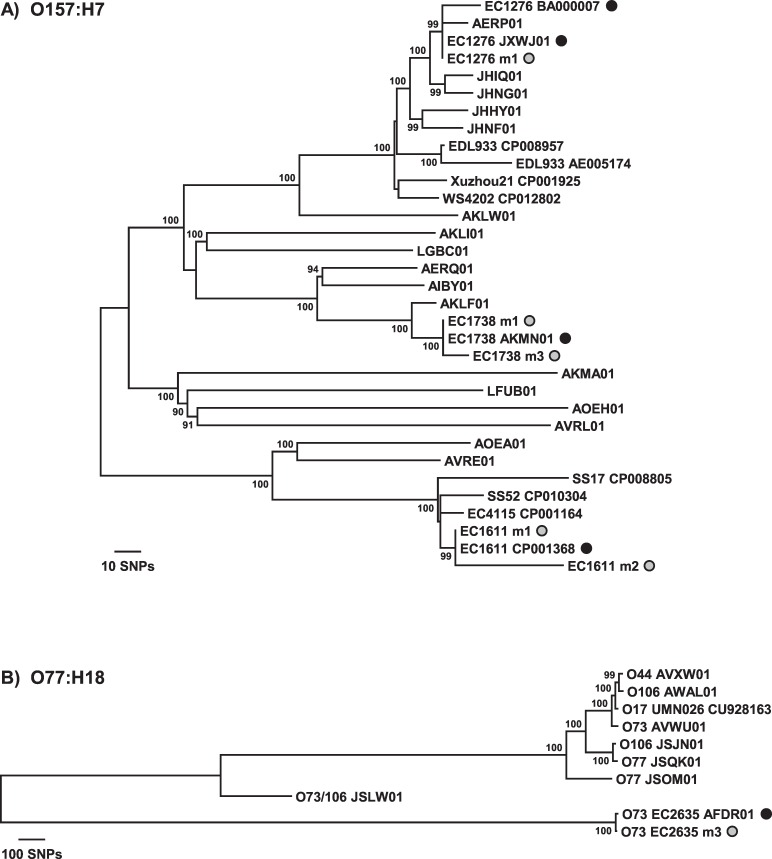
Finer-scale resolution of the phylogenetic relationships resulting from *E*. *coli* core gene SNP analysis. Neighbor-joining trees for the O157:H7 and O77:H18 clonal groups were constructed using a *p* distance matrix and 500 bootstrap replications. The spiked STEC strains and metagenomic samples are indicated by the black and gray circles, respectively. Bootstrap values >80% are given at the internal nodes.

**Fig 4:**
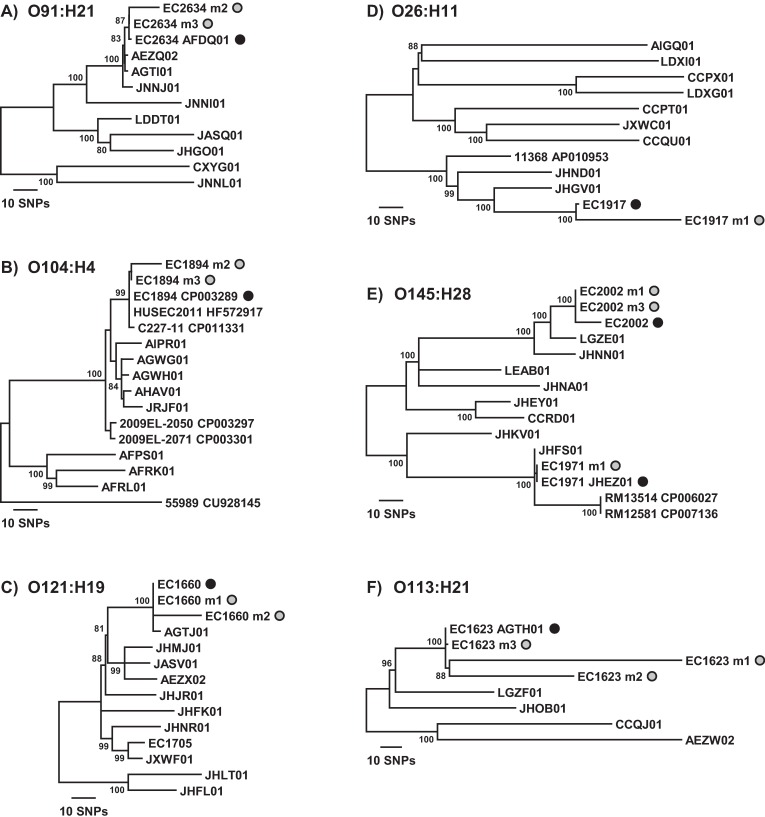
Finer-scale resolution of the phylogenetic relationships resulting from *E*. *coli* core gene SNP analysis. Neighbor-joining trees for the O91:H21, O104:H4, O121:H19, O26:H11, O145:H28, and O113:H21 clonal groups were constructed using a *p* distance matrix and 500 bootstrap replications. The spiked STEC strains and metagenomic samples are indicated by the black and gray circles, respectively. Bootstrap values >80% are given at the internal nodes.

In order to determine whether two different spinach samples are contaminated with the same STEC strain, or to compare a contaminating strain to a clinical isolate, it is necessary to examine strain relatedness on a finer scale. To this end, we investigated whether we could distinguish metagenomic samples within a subcluster of a particular *E*. *coli* serotype from each other and from other closely related strains. Additional *E*. *coli* genomes were included in the *E*. *coli* core gene SNP analysis to fill out the subclusters in which the spiked STEC genomes are found (Figs 3 and 4). Examination of the O157:H7 subcluster affirms that the metagenomic samples and respective spiked STEC genomes are nearest neighbors (Fig 3A). Although some metagenomic samples display nucleotide differences at SNP positions compared to the spiked STEC genome, the tree topology demonstrates that the strains are more closely related to each other than to any other *E*. *coli* O157:H7 strains. Of note, there are two genome sequences available in GenBank for EC1276 (Sakai), a closed genome (accession no. BA000007) and a more recent draft genome (accession no. JXWJ01). While sample EC1276m1 exhibits 15 mismatches at SNP positions in comparison to the older closed genome sequence, there are 0 mismatches between EC1276m1 and the newer draft genome sequence (Table 4, Fig 3A). The two metagenomic samples spiked with STEC O145:H28 strains can also be clearly distinguished from each other in a phylogeny at a fine scale and have the spiked strain genome as nearest neighbor (Fig 4E). The other serotype for which two STEC strains were used for spiking was O121:H19. While sample EC1705m1 clusters with the EC1705 genome on a coarser scale (Fig 2), the 142 mismatches at SNP positions were too great to include the sample in the O121:H19 tree on a finer scale because it would have distorted the resulting tree topology (Table 4, Fig 4C). This was also the case for sample EC1660m3, which contains indigenous *E*. *coli*. However, metagenomic samples EC1660m1 and EC1660m2 cluster with the EC1660 genome and are clearly differentiated from the other *E*. *coli* O121:H19 genomes in the subcluster.

Of the 24 samples clustering with the spiked genome in Fig 2, one other sample containing only the spiked *E*. *coli* strain, EC2635m2, and two other samples containing indigenous *E*. *coli*, EC1894m1 and EC2634m1, could not be included in the subcluster trees because the number of mismatches at SNP positions was high enough to cause topological distortion of the trees (Table 4, Figs 3B, 4A and 4B). Strains within the O17/O44/O73/O77/O106 group of O types share identical backbone O-subunit structures but exhibit multiple O-antigen types due to glucosylation of the common backbone, with the unmodified backbone exhibiting the O77 type [25]. As such, we refer to the clonal group containing the spiked STEC O73:H18 strain EC2635 as the O77:H18 group. Inspection of the O26:H11, O77:H18, O91:H21, O104:H4, and O113:H21 subclusters demonstrates that the SNP analysis performed on the shotgun metagenomic data assemblies allows clear differentiation of the samples from other *E*. *coli* strains in the subcluster and places the samples with the respective spiked STEC genome as nearest neighbor (Figs 3B, 4A, 4B, 4D and 4F).

A more detailed inquiry was performed regarding the SNP analysis results for some spiked samples not containing indigenous *E*. *coli*. This led to the discovery that in some cases a large percentage of the mismatches between the metagenomic assembly data and the spiked genome sequence occur in a single gene. For example, 37 of the 43 mismatches at SNP sites for sample EC1611m2 occur in the *secY* gene. A BLASTn search of the assembled metagenome for that sample using the *secY* gene sequence from *E*. *coli* strain K-12 MG1655 yields a contig that when used as a query in a BLASTn search of the NCBI database returns a best match to *Enterobacter cloacae* (GenBank accession number CP001918.1). Additional samples investigated that contained a high percentage of mismatches at SNP sites in single genes were EC1917m1, EC1623m2, and EC1705m1. The EC1917m1 assembly has 44 of 47 nucleotide differences in *rpoC*, the EC1623m2 assembly has 52 of 64 mismatches in *ptsG*, and the EC1705m1 assembly has 27 and 40 mismatches in *proV* and *secY*, respectively. The mismatches at SNP positions in the EC2635m2 metagenome are scattered over 377 genes.

We reasoned that using other assembly methods where only *E*. *coli* specific reads would be used for the core gene SNP analysis might alleviate this problem, yet wanted to continue analyzing the data in an agnostic fashion that assumed no *a priori* knowledge of a contaminating STEC strain. To test this hypothesis, the metagenomic datasets from samples EC1611m2, EC1623m2, EC1705m1, EC1917m1, and EC2635m2 were used in two different assembly strategies. First, the *E*. *coli* specific reads were binned and a *de novo* assembly using only those reads was performed, and second, the reads were used in a reference-based assembly using the genome for *E*. *coli* K-12 MG1655. SNP analysis determined the number of mismatches between the spiked genome and *E*. *coli* specific reads assembly and reference-based assembly, respectively, for each sample to be: EC1611m2, 69 and 58; EC1623m2, 125 and 55; EC1705m1, 1433 and 485; EC1917m1, 220 and 19; EC2635m2, 1192 and 1171. In every case, the reference-based assembly method resulted in fewer mismatches than the *E*. *coli* specific *de novo* assembly. However, the reference-based assembly resulted in a greater number of mismatches than the *de novo* assembly using all metagenomic reads for samples EC1611m2, EC1705m1 and EC2635m2 (Table 4). The only significant improvement was for sample EC1917m1, where the number of mismatches decreased from 47 to 19. Interestingly, rather than 44 mismatches located in *rpoC* and 1 mismatch located in each of 3 different genes, the reference-based assembly determined the 19 mismatches at SNP locations to be distributed among 12 genes, none of which was *rpoC*. In fact, the 55 mismatches determined from the reference-based assembly for EC1623m2 were dispersed over 24 genes, none of which was *ptsG*, compared to 7 genes for the metagenomic data assembly. Similarly, the 58 mismatches determined from the reference-based assembly for EC1611m2 were distributed among 15 genes, none of which was *secY*, compared to 4 genes for the metagenomic data assembly. Therefore, using the *E*. *coli* K-12 MG1655 reference-based assembly resulted in an improvement in that the mismatches at SNP sites were not clustered in one or a few genes, but the total number of mismatches increased in 3 of the 5 samples examined with this assembly method.

## Discussion

Utilizing shotgun metagenomic sequencing for both pathogen detection and characterization would expedite the time necessary to ascertain the risk level of STEC-contaminated fresh produce for public health. Moreover, rapid detection methods are particularly important with fresh produce because it has a short shelf life. Our results using a variety of genomically disparate STEC strains to spike bagged spinach reveal that after overnight enrichment of the contaminated sample, detection sensitivity comparable to or better than current PCR and qPCR methods can be attained [10]. The low experimental contamination level of 0.1 CFU/g used in this work allowed detection and virulence profiling in 34 of 36 (94%) of the samples when multiplexing 12 samples together for sequencing, yielding an average total read count of 2.4 million per sample. Even when only the forward read from the paired-end 250 bp reads was used for analysis, the molecular serotype and virulence genes of the contaminating STEC strain could be correctly determined. This is significant as it would decrease the sequencing time from approximately 40 hours to 22 hours. The result that virulence characterization of the spiked STEC was not achieved for 2 of the 36 samples indicates, however, that a practical limit of more than 2.4 million reads (2 x 250 bp) would be necessary if a lower false negative rate is desired. While the present experiments were performed using the same brand of spinach, experiments using the STEC O157:H7 strain Sakai to spike six different brands of spinach demonstrate that this level of sensitivity can be achieved with a variety of packaged ready-to-eat spinach samples (data not shown).

It was not surprising to discover that some of the bagged spinach samples contained indigenous *E*. *coli* as surveys in the United States and other countries have shown that it is not an uncommon occurrence [2], with prevalence rates of 8.9% and 13% reported for the United States [28, 29]. The 61% prevalence rate determined from our results cannot be directly compared to the reports mentioned above as our experiments included an enrichment step which would favor the detection of very low levels of indigenous *E*. *coli*. With the exception of O26, the serogroups discovered on the bagged spinach do not match any of the STEC serogroups reportedly isolated over a ten year period by the Microbiological Data Program from spinach or other selected produce by conventional methods [2]. Analysis of the sequence data for samples EC2634m1 and EC2635m1 revealed that both contained an *E*. *coli* having serogroup O26 among others, and *stx1a*, *eae*, and *ehxA* along with the H11 *fliC* allele (Table 2). The spinach used for these samples was taken from different bags procured at the same time. Another serogroup considered to be linked to human illness [15], O45, was detected along with serogroup O101 in one sample. *E*. *coli* O45 and O101 were identified together previously in an unspiked spinach sample, and in that case it was determined that neither strain carried a Shiga toxin gene [16]. Also noteworthy, *stx1a* was detected in sample EC1611m1 (Table 2) but no O or H types other than O157:H7 of strain EC1611 were detected. The number of reads mapping to *stx1a* compared to the *stx2* genes is significantly lower, suggesting the genes do not belong to the same genome. It is possible that the spinach in that sample was already contaminated with an STEC O157:H7 strain containing *stx1a*. Alternatively, the *stx1a*-positive strain could possess a molecular serotype not represented in our database or the serotyping loci were not observed due to the stochastic nature of genome coverage at low levels. The discovery of more than one *E*. *coli* strain in some spinach samples exemplifies the inherent complexity of detecting pathogens using metagenomic data. Specifically, while attributes such as species, serotype, or virulence genes can be easily detected, deconvoluting the data to definitively associate these with the same genome cannot be done without further analysis.

Experimental variability was expected in the growth of different spiked STEC strains during enrichment given their differences in growth in pure culture compounded by competition within communities inherent on the spinach samples. 16S rRNA and/or shotgun metagenomic sequencing has been used to determine the abundances of bacterial species on pre-enriched samples as well as to determine shifts in the microbial community during enrichment for foodborne pathogens such as *Salmonella enterica* on tomatoes [30] and cilantro [31] and STEC on spinach [16]. It is clear from these studies that the pathogen is growing amidst a dynamically changing microbial community structure as the enrichment proceeds. The FDA BAM protocol used for STEC enrichment from leafy green produce [18] was designed to favor growth of Enterobacteriaceae. Our result demonstrating that >90% of the microbial community consists of Enterobacteriaceae for all 36 samples is consistent with the intent of that protocol (Fig 1). Percent abundance results of the spiked STEC strain in the biological replicate samples reveals significant variation in the STEC’s ability to compete with other Enterobacteriaceae during enrichment, including indigenous *E*. *coli* strains. This suggests that the bagged spinach samples have variation in their initial microbial community compositions as small differences in pre-enrichment abundances of Enterobacteriaceae may be magnified during enrichment. The differences in abundances between replicate samples for a given spiked STEC strain were just as great in magnitude as the inter-strain differences. The two strains EC1705 and EC2635 each produced one sample of percent abundance below 10% even when the spiked strain was the only *E*. *coli* strain present (Fig 1). Of note, although the spiked STEC strains in samples EC1705m2 and EC2635m1 appear to be detected in the microbial community analysis, the serotype and virulence gene profile could not be determined (Table 2). Thus, the STEC strains were growing but not well enough to have sufficient genome coverage for virulence gene detection given the number of total sequence reads (1.79 and 2.18 million for EC1705m2 and EC2635m1, respectively). These results emphasize the need to establish confidence intervals for false-negatives given a particular sequencing depth.

Performing spiking experiments not only allows for establishing detection sensitivity, but also evaluation of consistency in results between the types of analyses performed since the genomic information for the contaminating strain is known. A comparison of the percent abundances of the spiked STEC strain and the indigenous *E*. *coli* strain(s) can be made for samples in which they belong to different *E*. *coli* phylogroups (Fig 1). We have also determined read counts mapping to the different serotyping loci (Table 2 for spiked strains, data not shown for indigenous strains) thus are able to infer which strain is more abundant. The results obtained from these two methods of determining the comparative abundance of spiked versus indigenous *E*. *coli* strain(s) in the samples are in agreement. Two methods were also used to quantify the portion of the sample that can be attributed to the spiked STEC strain for samples without indigenous *E*. *coli*, that is, microbial community analysis (Fig 1) and mapping reads to the respective STEC genome (Table 3). The same quantitative trend is observed from sample to sample for both methods, however, the percent mapped reads is similar to or lower than the percent abundance of the *E*. *coli* phylogroup of the spiked STEC identified in the community analysis for all samples except EC1705m1 and EC1660m2. This result was also observed in our previous work [16]. Two factors may contribute to the quantitation discrepancy between the methods: (i.) the stringent parameters used for mapping underestimates the percent spiked STEC-associated reads and (ii.) reads arising from members of the microbial community that are not included in the *k*-mer database used for analysis, i.e. unidentified bacteria or not of bacterial origin, cause an overestimation of the percent abundances of all the identified bacterial species (which would include the spiked STEC strain) by decreasing the denominator which includes only identified reads.

Whole genome sequencing (WGS) is a valuable and effective tool in characterizing pathogens during an outbreak investigation as it provides superior resolution for strain discrimination than other subtyping methods currently used [11, 32, 33]. These real-time outbreak investigations and other retrospective studies [34, 35] have demonstrated the utility of WGS in linking clinical and foodborne isolates with epidemiological relevance. However, they rely on pathogen isolation before sequencing. The ability to use shotgun metagenomic data to infer strain relatedness would decrease outbreak response time. Applying our *E*. *coli* core genome SNP analysis to samples without indigenous *E*. *coli* on the spinach demonstrates the potential to infer fine-scale phylogenetic relatedness of a contaminating STEC strain to other *E*. *coli* (Figs 2, 3 and 4). In fact, for samples without indigenous *E*. *coli* that have spiked strain genome coverages of >30x, the number of mismatches between the assembly and the spiked STEC genome at *E*. *coli* core gene SNP sites is ≤21 with the exception of sample EC1611m2 (Tables 3 and 4). Although the genome coverage for sample EC1611m2 was 50x (Table 3), community analysis identified *E*. *cloacae* as present in the enriched sample (Fig 2) and, as determined from the BLASTn analysis, the *secY* gene for the spiked STEC EC1611 was absent from the EC1611m2 assembly. This problem was not encountered using a reference-based assembly approach; however the total number of mismatches between the EC1611 genome and the EC1611m2 assembly increased from 43 to 58. The very low coverages of 16x and 5x for EC1705m1 and EC2635m2, respectively, would be predicted to affect the performance of accurate SNP analysis even for pure isolates. Thus, the higher number of mismatches determined for those samples is not unexpected. While molecular serotyping and virulence characterization was possible for these two samples (Table 2), deeper sequencing is necessary to perform more accurate *E*. *coli* SNP analysis.

Our results demonstrated that phylogenetic relatedness was attainable with high resolution for a contaminating STEC strain when it is in much greater abundance than indigenous *E*. *coli* strains after enrichment, such as for samples EC1660m1, EC1971m1, and EC2002m3 (Figs 1 and 4, Table 4). As expected however, for samples in which the indigenous *E*. *coli* strain grew to a greater abundance than the spiked STEC strain, SNP analysis performed on the metagenomic data leads to a bias towards the most abundant indigenous *E*. *coli* strain in the resulting *E*. *coli* phylogeny. Examples of this are samples EC1276m2, EC1705m2, EC1738m2, EC1971m2, EC1971m3, EC2002m2, and EC2635m1. The phylogroup of the most abundant indigenous *E*. *coli* is the phylogroup the sample clusters with from the SNP analysis for those samples (Figs 1 and 2). Clearly, when using metagenomic sequence data, it is important to first determine whether there is more than one *E*. *coli* serotype present before relying on SNP analysis results with confidence when comparing the relatedness of a contaminating STEC strain with other *E*. *coli* strains.

## Conclusion

This study explores the practicality of using shotgun metagenomic sequencing to detect and characterize STEC strains contaminating fresh bagged spinach at a very low level of 0.1 CFU/g. Using biological replicate samples of spinach spiked with a variety of pathogenic STEC strains, our bacterial community analysis results reveal extensive variation in growth of the spiked STEC after overnight enrichment between replicate samples and between different STEC strains. This is at least in part due to the presence of indigenous *E*. *coli* strains in some bagged spinach samples. We demonstrated that while the presence of indigenous *E*. *coli* did not have a substantial impact on the ability to detect the virulence genes and serotyping loci of the spiked STEC strain, it did significantly affect determination of strain-level relatedness of the contaminating STEC strain to other *E*. *coli* strains using core gene SNP analysis. Our results underscore the need for methods to bioinformatically separate the genomes of two *E*. *coli* strains included in a microbial community. This study demonstrates, however, that when indigenous *E*. *coli* strains are not present on the spinach, SNP analysis based on metagenomic sequence data is effective in clearly differentiating the spiked STEC strain from other related STEC strains. As with detection, the SNP analysis results would be enhanced for some samples that had low STEC genome coverage by multiplexing fewer samples on a sequencing run. Future studies on adjustments to the enrichment protocol may also allow for greater detection sensitivity. In all, the results of this work highlight the value of utilizing a shotgun metagenomics approach for detection and strain level characterization of STEC strains contaminating fresh produce as it is possible to obtain significantly more information about a contaminating strain in a shorter time period than with conventional methods. In addition, this work may provide valuable information for constructing and assessing bioinformatic pipelines that will need to be created for analysis of shotgun metagenomic data generated from produce samples possibly contaminated with STEC.
